# A Broad-Band Self-Powered Photodetector Based on a MoTe_2_/Bi_2_Te_3_ Heterojunction for Optical Imaging and Bias-Controlled Signal Modulation

**DOI:** 10.3390/ma19061270

**Published:** 2026-03-23

**Authors:** Shaoxiong Du, Kunle Li, Weijie Li, Jiahui Feng, Yunwei Sheng, Lili Tao, Zhaoqiang Zheng, Wei Song, Yu Zhao

**Affiliations:** 1 Guangdong Provincial Key Laboratory of Functional Soft Condensed Matter, School of Material and Energy, Guangdong University of Technology, Guangzhou 510006, China; dushaoxiong0402@outlook.com (S.D.); qq1060338341@163.com (K.L.); 13169965646@163.com (W.L.); fengjhui@163.com (J.F.); taoll@gdut.edu.cn (L.T.); zhengzhq5@gdut.edu.cn (Z.Z.); 2 Analysis and Test Center, Guangdong University of Technology, Guangzhou 510006, China; songw@gdut.edu.cn

**Keywords:** MoTe_2_, Bi_2_Te_3_, van der Waals heterostructure, broad spectral response, self-powered photodetector

## Abstract

Self-powered photodetectors are highly demanded in applications but often suffer from limited spectral absorption, slow response speed, and high dark currents. Two-dimensional van der Waals heterostructures have emerged as promising candidates owing to their designable structures and excellent performance. Herein, we construct a MoTe_2_/Bi_2_Te_3_ heterostructure and investigate its photoelectric properties. At zero bias, it exhibits a broad photovoltaic response ranging from 405 to 1550 nm. Benefiting from the interfacial built-in electric field, it achieves a responsivity of 1.38 A/W and a detectivity of 1.90 × 10^12^ Jones at 532 nm and retains 174.56 mA/W and 2.4 × 10^11^ Jones at 1060 nm, together with a low dark current of 1.6 × 10^−12^ A. Upon a reverse bias of −1 V and 532 nm laser illumination at an intensity of 19.0 W/m^2^, the responsivity is further boosted to 36.22 A/W, accompanied by rise and decay times of 32 ms and 33 ms, respectively. Taking advantage of the distinct optical switching ratios at zero/non-zero biases, application in optical imaging and bias-controlled signal modulation is realized, highlighting the heterojunction’s potential as a broadband self-powered photodetector.

## 1. Introduction

Photodetectors occupy a pivotal position in numerous civilian and military technologies, including spectral analysis, communications, and night-vision imaging [1,2,3,4,5]. However, conventional photodetectors rely on external power supplies, which limit their application in energy-autonomous intelligent sensing systems. Therefore, self-powered photodetectors that enable weak-signal detection without external bias have attracted considerable attention [6,7]. To meet the demand for broadband and fast-response photodetectors, two-dimensional transition metal dichalcogenides (2D TMDs) have been widely investigated [8,9,10,11,12]. Nevertheless, photodetectors based on single TMD materials suffer from inherent drawbacks, such as insufficient light absorption, slow response speed, and high dark current [13,14,15,16,17]. Constructing van der Waals (vdW) heterostructures provides an effective strategy to address these issues. Such heterostructures can avoid lattice-matching limitations and form atomically sharp interfaces while retaining the intrinsic properties of individual materials and inducing novel interfacial coupling effects. They thus offer an ideal platform for fabricating high-performance self-powered photodetectors [18,19,20,21].

Currently, progress has been made in novel self-powered photodetectors with extended spectral range based on 2D heterostructures. For instance, Li et al. [22] reported a high-performance, self-powered photodetector based on a NbSe_2_/MoS_2_ heterostructure, achieving a spectral response from 405 to 980 nm, with a high responsivity of 455.3 mA/W and a detectivity up to 1.9 × 10^12^ Jones. Similarly, Che et al. [23] constructed a highly sensitive broadband phototransistor based on PtSe_2_/MoSe_2_, with a detection capability ranging from 532 to 1550 nm, but it still requires an external bias to operate.

Bismuth telluride (Bi_2_Te_3_) is a typical n-type topological insulator with a layered structure [24]. The topologically protected surface states of Bi_2_Te_3_ enable high carrier mobility and excellent ambient stability. With a narrow bulk bandgap of approximately 0.15 eV [25], Bi_2_Te_3_ exhibits a theoretical infrared absorption edge up to 8.2 μm, making it a promising material for infrared photodetection [26]. While integrating Bi_2_Te_3_ with p-type TMD, semiconductors can significantly broaden the spectral response of the heterostructure devices; its extremely narrow bandgap also introduces challenges. Upon absorbing photons of higher energy than the bandgap, carriers are excited to the conduction band minimum via non-radiative transitions, with excess energy converted into lattice vibrations, which increases the dark current and leads to performance degradation, especially at high optical power levels [27,28]. Zhao [29] et al. designed an asymmetric Te/Bi_2_Te_3_/In_2_O_3_ heterojunction structure whose infrared photodetector exhibited excellent self-powered photodetection performance at 850, 980, and 1050 nm, achieving a responsivity as high as 0.4195 mA/W under 850 nm laser illumination. Yang [30] et al. fabricated a Bi_2_Te_3_/Si heterojunction photodetector, which exhibited a high responsivity of 16.44 mA/W under 470 nm illumination, a high specific detectivity of 2.44 × 10^11^ Jones, and fast rise/decay times of 11/13 ms. Wu [31] et al. developed a self-powered photodetector based on a Bi_2_Te_3_/Sb_2_O_3_/p-Si vertical heterojunction, which exhibited responsive performance over a broad UV–vis–NIR range from 254 to 1050 nm with a high responsivity of 316.5 mA/W, a specific detectivity of 6.19 × 10^11^ Jones, and fast rise/decay times of 24.6/25.1 ms. Improved performance in infrared detection applications has been demonstrated, illustrating the effectiveness of structure design in optimizing carrier dynamics and noise suppression. Similarly, molybdenum ditelluride (MoTe_2_) is established as one of the leading candidate materials for optoelectric heterojunctions owing to its narrow bandgap of approximately 1 eV, along with high atmospheric stability and superior carrier mobility for both electrons and holes [32].

In this work, we investigate the optoelectrical properties of MoTe_2_/Bi_2_Te_3_ p-n heterojunction fabricated via mechanical exfoliation and dry transfer, hopefully to construct a heterostructure of broad photoresponse with the help of wide-spectral absorbing Bi_2_Te_3_ and dark-current suppressing MoTe_2_. Electrical measurements reveal pronounced rectification behavior in dark and clear photovoltaic effect under illumination with a broad spectral response ranging from 405 to 1550 nm. It shows a dark current as low as 2 × 10^−12^ A, a photocurrent switching ratio (*I*_light_/*I*_dark_) larger than 10^3^, a responsivity of 1.38 A/W, and a detectivity of 1.90 × 10^12^ Jones at zero drain bias (*V*_ds_) using a 532 nm laser. The excellent self-powered performance can be maintained at near-infrared 1060 nm with a responsivity of 174.56 mA/W and a detectivity of 2.4 × 10^11^ Jones. To showcase the great application potential of these self-powered photodetectors, room-temperature optical imaging and ASCII code transmission with bias-controlled signal modulation are reliably realized. The excellent performance of the MoTe_2_/Bi_2_Te_3_ heterojunction ranks top among the state-of-the-art for self-powered photodetectors based on 2D semiconductor heterostructures.

## 2. Materials and Methods

### 2.1. Device Fabrication

MoTe_2_/Bi_2_Te_3_ van der Waals heterostructure (vdWH) transistors were fabricated by stacking the mechanically exfoliated thin flakes of MoTe_2_ (99.999%, six Carbon Technology, Shenzhen, China) and Bi_2_Te_3_ (99.999%, six Carbon Technology, Shenzhen, China). Bulk MoTe_2_ crystals were thinned through iterative folding-and-peeling processes using polyvinyl chloride (PVC) tape (Ultron Systems, UST, Moorpark, CA, USA). The PVC tape with exfoliated MoTe_2_ flakes was gently pressed at room temperature for 20 s, and the flakes were then transferred onto a polydimethylsiloxane (PDMS) stamp via rapid vertical peeling. The PDMS stamp was subsequently thermally laminated onto a SiO_2_/Si substrate at 80 °C for 30 s. After rapid vertical peeling, the flakes were firmly adhered to the substrate surface. Flakes with a lateral size > 25 μm and clean surfaces were selected for subsequent experiments.

The MoTe_2_/Bi_2_Te_3_ vdWH was assembled via a standard dry-transfer technique. First, the PDMS stamp was adhered to a clean glass slide, and a thin polyvinyl alcohol (PVA) film was spin-coated on the PDMS surface. The assembly was baked at 54 °C for 4 min to remove residual moisture. Subsequently, the PDMS/PVA stamp was brought into contact with the SiO_2_/Si substrate bearing exfoliated MoTe_2_ flakes, allowing the flakes to adhere to the PVA film. Another SiO_2_/Si substrate with the target Bi_2_Te_3_ flakes was placed on a micromanipulator-equipped transfer stage (IT-800, IVTEST Technology, Guangzhou China) and adjusted beneath the stamp. Under an optical microscope, the MoTe_2_ flake was precisely aligned with the Bi_2_Te_3_ flake. The stamp was slowly lowered until the two flakes were fully laminated with no visible trapped bubbles in the contact area. The assembly was then heated at 90 °C for 3 min to obtain the MoTe_2_/Bi_2_Te_3_ heterostructure. Subsequently, to remove the PVA sacrificial layer, the substrate was immersed in deionized water at 55 °C for 9 min until the PVA was completely dissolved. To eliminate interfacial contamination introduced during the transfer process, the assembled heterostructure was annealed at 200 °C for 1 h under a continuous flowing nitrogen (N_2_) atmosphere. Ultimately, the source and drain electrode regions were patterned via ultraviolet (UV) photolithography, and titanium/gold (10 nm Ti/60 nm Au) metal contacts were deposited via thermal evaporation to complete the device fabrication.

### 2.2. Device Characterization

The morphology, composition, and microstructure of the materials were analyzed using various techniques, including an optical microscope (BA310MET, Motic, Xiamen, China), atomic force microscopy (AFM) with a Bruker Dimension FastScan, and X-ray photoelectron spectroscopy (XPS, Thermo Fisher, Waltham, MA, USA, Escalab 250Xi). The vacuum level within the analysis chamber exceeds 5.0 × 10^−10^ mbar. During the analysis process, the beam spot of the X-ray source (Al target) measures 650 μm, with operating voltages and currents of 15 kV and 15 mA, respectively, and Raman spectroscopy (NOST, FEX, with a 532 nm excitation laser). Electrical and photoelectric properties were measured using a Keithley2636B source meter (KEITHLEY, Cleveland, OH, USA), with the Bi_2_Te_3_ source side grounded. The photo response was measured in air at room temperature under 405, 532, 635, 808, 1060, 1310, and 1550 nm lasers, respectively.

## 3. Results and Discussion

**Figure 1:**
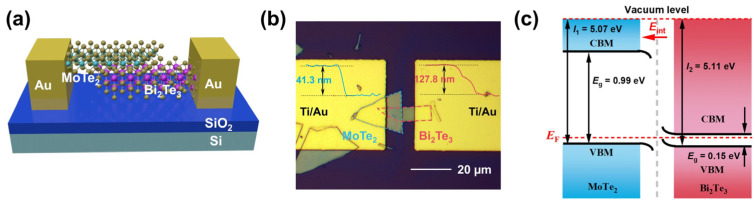
(**a**) Schematic diagram of a van der Waals heterojunction of MoTe_2_/Bi_2_Te_3_. (**b**) Optical image of the MoTe_2_/Bi_2_Te_3_ device, where the insets are the height profiles characterized by atomic force microscopy. (**c**) Energy band diagrams of MoTe_2_ and Bi_2_Te_3_ after contact. *E*_F_ is the Fermi energy level, and *E*_int_ is the internal built-in electric field of the heterojunction.

Figure 1a presents a schematic three-dimensional front view of the MoTe_2_/Bi_2_Te_3_ device with the MoTe_2_ flake on top of the Bi_2_Te_3_ flake, where the Ti and Au are used as drain and source contacts, and a heavily doped silicon substrate with a 300 nm SiO_2_ layer as the back gate. An optical microscopy image of the fabricated MoTe_2_/Bi_2_Te_3_ heterostructure is shown in Figure 1b, where the insets indicate the thicknesses of approximately 41.3 nm for the MoTe_2_ flake and 127.8 nm for the Bi_2_Te_3_ flake. The p-type and n-type semiconductor characteristics of MoTe_2_ and Bi_2_Te_3_, respectively, are revealed by their transfer curves in Figure S1 in the Supplementary Materials. The band position of the two constituent materials is important for the determination of the band alignment of the heterostructure. Here, the valence-band maximum (VBM, denoted by *E*_v_) of the two materials was obtained by linearly extrapolating the low binding energy region of the XPS spectra to the baseline (Figure S2a,b). With the bandgap values of 0.99 eV and 0.145 eV obtained by first principle calculation based on density function theory (DFT) for MoTe_2_ and Bi_2_Te_3_, respectively, the conductance-band minimum (CBM) with respect to VBM is able to be determined. Finally, the Kelvin probe force microscope (KPFM) used to measure the surface potential is able to acquire the work function difference between MoTe_2_ and Bi_2_Te_3_, by which a Fermi energy level difference of 60.2 mV for the two materials before contact can be obtained. When the heterojunction is formed by contacting MoTe_2_ and Bi_2_Te_3_, the Fermi levels in the two materials are aligned under thermal equilibrium, which results in type-II band alignment, as illustrated in Figure 1c. Such band alignment can facilitate the separation of photo-induced electron-hole pairs, which is beneficial to the construction of efficient photodetectors. A detailed analysis of XPS, KPFM, and calculation results to obtain the band structure can be seen in the SI and Figure S2.

**Figure 2:**
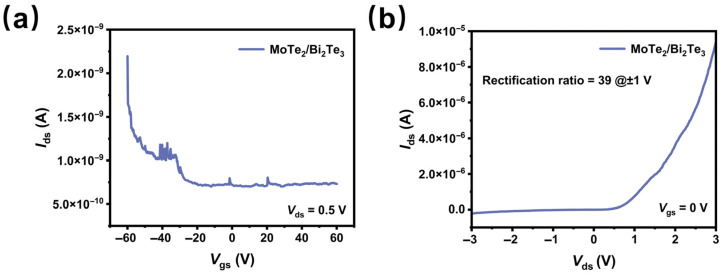
(**a**) Transfer curves measured at *V*_ds_ = 0.5 V of the MoTe_2_/Bi_2_Te_3_ device. (**b**) The *I*_ds_–*V*_ds_ output curve measured at *V*_gs_ = 0 V of the MoTe_2_/Bi_2_Te_3_ device, where the rectification ratio is calculated using the *I*_ds_ values at forward and reverse biases ±1 V.

Figure 2a shows the wide-range transfer curve of the MoTe_2_/Bi_2_Te_3_ device, which reveals the p-type behavior of the heterostructure. As can be seen in Figure 2b, the heterostructure shows a rectification ratio of approximately 39 at *V*_ds_ = ±1 V, indicating a clear diode characteristic. This rectification behavior is a typical characteristic of a pn junction. Namely, the drain current is large when the heterostructure is under forward bias (p-type MoTe_2_ under positive bias) and is small under reverse bias. The XPS spectra with corresponding quantitative fitting data and Raman spectra confirm the chemical and phase composition of pristine MoTe_2_ and Bi_2_Te_3_, as well as the successful fabrication of the MoTe_2_/Bi_2_Te_3_ heterostructure. Detailed results are provided in Figures S3 and S4, and Table S1 of the Supplementary Materials. The AFM image showing the surface morphology of the individual material is included in Figure S5, where white spots are observed on the surfaces of the MoTe_2_ and Bi_2_Te_3_ materials, indicating the surface oxidation as well.

**Figure 3:**
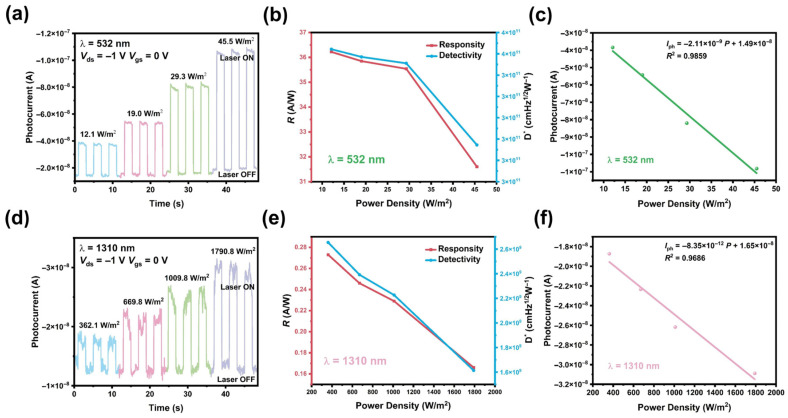
The photoresponse of the MoTe_2_/Bi_2_Te_3_ heterojunction at *V*_ds_ = −1 V and *V*_gs_ = 0 V in dark and under the irradiation of (**a**) 532 nm and (**d**) 1310 nm laser, respectively, with different light power intensities. The light-power-dependent responsivity and detectivity under the irradiation of (**b**) 532 nm and (**e**) 1310 nm lasers, respectively. Photocurrent of the heterojunction as a function of incident power density at (**c**) 532 nm and (**f**) 1310 nm laser wavelengths, respectively, where the symbols are the experimental results and solid lines are the linear fits.

The photoresponse of the MoTe_2_/Bi_2_Te_3_ device is systematically evaluated under different power intensities and different drain-source voltages (*V*_ds_). Figure 3a,d present the time-dependent current results (*I*_ds_-*t*) obtained at *V*_ds_ = −1 V in periodic dark/illumination cycles, using a laser of 532 and 1310 nm at different powers. The curves exhibit prominent and repeatable switching characteristics, demonstrating the device’s reliable response to all four wavelengths across various power intensities. Key parameters of responsivity ($R_{\lambda }$) and detectivity ($D^{*}$) are calculated by the values extracted from Figure 3a,d and summarized in Figure 3b,e. Responsivity refers to the photocurrent generated per unit power of incident light, expressed as Equation (1):

(1) $$R_{\lambda }=\frac{I_{ph}}{P_{\lambda }S}$$

where $I_{ph}$ is the net photocurrent, defined as the difference in light current and dark current at the same *V*_ds_, that is, $I_{ph}=I_{light}-I_{dark}$; $P_{\lambda }$ is the incident light power density; and S is the effective illuminated working area of the heterojunction. Detectivity is used to characterize the sensitivity of the photodetector, reflecting its ability to detect low-level signals, written as Equation (2):

(2) $$D^{*}=\frac{R_{\lambda }\sqrt{S}}{\sqrt{2eI_{dark}}}$$

where $I_{dark}$ is the dark current, and *e* is the elemental charge. The MoTe_2_/Bi_2_Te_3_ device achieves impressive performance under 532 nm laser irradiation of 12.1 W/m^2^, with the maximum $R_{\lambda }$ reaching 36.22 A/W and $D^{*}$ reaching 3.52 × 10^11^ Jones. The device maintains a high responsivity of 273 mA/W and a high detectivity of 2.65 × 10^9^ Jones under 1310 nm illumination of 362.1 W/m^2^. Both the $R_{\lambda }$ and $D^{*}$ decrease when the light intensity increases, meaning that the photocurrent is saturated at low power intensity. To quantify the relation between the photocurrent and the laser power, linear fit of $I_{ph}=aP+b$ is used to describe the effect as shown in Figure 3c,f, where the good linear dependence of $I_{ph}$ on power demonstrates its potential as a linear photodetector. The *I*_ds_-*t* characteristics, corresponding $R_{\lambda }$ and $D^{*}$ values and fitting results at other wavelengths are provided in Supplementary Materials Figures S6–S8. Owing to the narrow bandgap of Bi_2_Te_3_ and the stable MoTe_2_, the MoTe_2_/Bi_2_Te_3_ heterostructure is expected to have a broad response range with suppressed dark current. Indeed, the MoTe_2_/Bi_2_Te_3_ device exhibits a clear photoresponse from 405 nm up to 1310 nm at −1 V bias, demonstrating the broadband response from the visible range to the near-infrared region.

**Figure 4:**
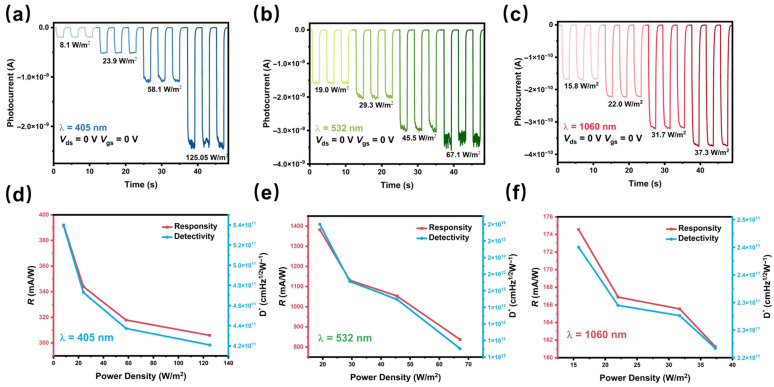
The photovoltaic response of the MoTe_2_/Bi_2_Te_3_ heterojunction at *V*_ds_ = 0 V and *V*_gs_ = 0 V in dark and under the irradiation of (**a**) 405 nm, (**b**) 532 nm, and (**c**) 1060 nm laser, respectively, with different light power intensities. The light-power-dependent responsivity and detectivity under the irradiation of (**d**) 405 nm, (**e**) 532 nm, and (**f**) 1060 nm, respectively.

Under zero external bias (*V*_ds_ = 0 V, *V*_gs_ = 0 V), the MoTe_2_/Bi_2_Te_3_ heterojunction operates in photovoltaic mode with an ultra-low dark current of 0.9 pA. As can be seen in Figure 4a–c, the device shows stable response under 405, 532, and 1060 nm at different power intensities, where the strongest photoresponse occurs under 532 nm illumination at a light intensity of 19.0 W/m^2^, as well, yielding a photocurrent switching ratio exceeding 10^3^. As correspondingly shown in Figure 4e, the calculated maximum $R_{\lambda }$ = 1.38 A/W, $D^{*}$ = 1.90 × 10^12^ Jones, which are remarkable values as one of the best reported self-powered photodetectors based on 2D heterojunctions [33]. As shown in Figure 4f, the photocurrent under zero bias at 1060 nm still exhibits an impressive value with the maximum responsivity of 174.56 mA/W and detectivity of 2.4 × 10^11^ Jones. The photovoltaic response of the MoTe_2_/Bi_2_Te_3_ heterojunctions at different powers across other wavelengths, along with the corresponding $R_{\lambda }$ and $D^{*}$ values, are contained in Supplementary Materials Figures S9 and S10. It is worth noting that the device exhibits photoresponse at 1550 nm under zero bias, as shown in Figure S10, manifesting distinct switching behavior, although inapparent, which verifies that the heterojunction can function as an expanded band self-powered photodetector. To further compare the photoresponse of the heterojunction device and the single MoTe_2_ device, their photocurrent and response speed are displayed in Figure S11.

**Figure 5:**
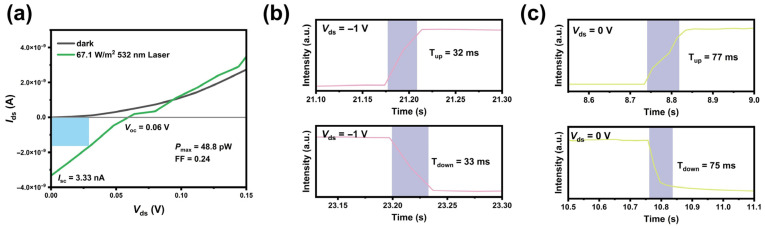
(**a**) *I*_ds_-*V*_ds_ curves at zero gate voltage, in dark and under 532 nm laser irradiation, respectively. Optical response times of the MoTe_2_/Bi_2_Te_3_ heterojunction under 532 nm illumination with an optical power density of 19.0 W/m^2^ at (**b**) *V*_ds_ = −1 V and (**c**) *V*_ds_ = 0 V, respectively.

From the *I*-*V* curve measured under a 532 nm laser illumination at 67.17 W/m^2^ in Figure 5a, the open-circuit voltage (*V*_oc_) and the short-circuit current (*I*_sc_) are obtained as 0.06 V and 3.33 nA, respectively. The output electrical power (*P*_el_) is given by the following Equation (3):

(3) $$P_{el}=V_{oc}I_{sc}$$

The fill factor (*FF*), a key parameter for evaluating the light conversion efficiency of photovoltaic devices, is defined as Equation (4):

(4) $$FF=\frac{P_{max}}{P_{el}}$$

The maximum output power *P*_max_ is 48.8 pW (at 0.029 V), marked by the light blue rectangular area in Figure 5a. The *FF* is deduced as 0.24, which is not that remarkable due to the low *V*_oc_. The response speed determines the application scenarios of photodetectors, generally defined as the time for the photocurrent to rise to 90% and fall to 10% of its maximum photocurrent. The rise and fall times of the heterojunction at *V*_ds_ = −1 V are obtained as around 32 ms and 33 ms, respectively, by the detailed *I*_ds_-*t* curves in dark and under 532 nm light, as shown in Figure 5b. The slower response speed of our device compared with other recently reported heterojunction devices is possibly attributed to the presence of TeO_2_ and MoO_x_ phases on the sample surface. As detailed in Table S1 of the Supplementary Materials, these oxide phases can introduce abundant deep-level trap states within the band gap. Acting as carrier trap centers, they effectively capture photogenerated carriers and significantly prolong their relaxation time, thereby retarding the rise and decay dynamics of the photocurrent [34,35]. Furthermore, the thick film exhibits a higher defect density than the ultra-thin single-crystalline film, and the increased trap density is accompanied by a drastic rise in trap capacitance. This trap capacitance is connected in parallel with the total capacitance of the device, which elevates the overall capacitance and, thus, increases the RC time constant. This gives rise to a pronounced tailing effect in the photoresponse and ultimately leads to the prolonged response time of the device [36]. As shown in Figure 5c, the response speed is nevertheless slower than that under applied drain bias, since carriers require a longer transit time to travel across the channel without the driving advantage of an external bias.

Moreover, the trap centers and/or defects in the individual materials, particularly at the interface, tend to accelerate the recombination of electron-hole pairs, reduce the carrier lifetime, and degrade the photoresponse characteristics, including the response speed. In addition, the space-charge region forms through carrier diffusion and drift equilibrium at the interface, generating a localized built-in field. In other words, the space charge effects accompanied by the type-II band alignment help to suppress the carrier recombination by driving them away from trap-rich regions to the circuits. Therefore, self-powered photodetectors of broad-spectral response are still obtained in this work. Table S2 compares the key performance metrics of the device in this work with those of previously reported heterojunction-based self-powered photodetectors. The MoTe_2_/Bi_2_Te_3_ photodetector exhibits a superior responsivity (*R*_λ_) and broader spectral response range than the reported counterparts, demonstrating great application potential as a promising candidate for next-generation self-powered optoelectronic devices. Further enhancement in performance could be achieved by using a high-quality crystal to fabricate devices with an atomically transparent interface and optimizing the band structure.

**Figure 6:**
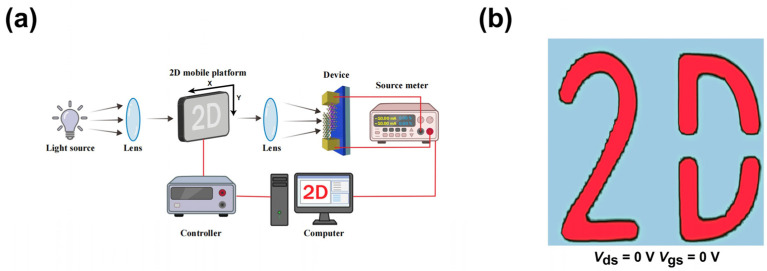
(**a**) Optical imaging system of the MoTe_2_/Bi_2_Te_3_ heterojunction device. (**b**) Current mapping results of “2D” pattern imaging using a 532 nm laser at *V*_ds_ = 0 V.

The distinct switching ratio at low bias and zero bias of the MoTe_2_/Bi_2_Te_3_ heterojunction suggests its great potential in low-power/self-powered operation imaging applications. A self-mounted imaging system is employed to explore this optical imaging capability, as illustrated in Figure 6a, consisting of a laser, a shadow mask, a source-meter unit, and a programmable motorized stage for movement along the X- and Y-axis. As the mask of the “2D” pattern between the laser and the photodetector translates horizontally along the X/Y directions, the incident light passes through the apertures of the mask and partially illuminates the device, generating a photocurrent. By recording the photocurrent at each pixel location, a high-resolution “2D” mapping image is reconstructed by computer processing, mimicking the shape of the shadow mask. To evaluate the self-driven imaging capability, the experiment is conducted at zero bias using a 532 nm laser, and the mapping image is presented in Figure 6b. It accurately captures the pattern profile of the mask with well-defined contrast against the background, demonstrating the inherent imaging capability of the device and its potential for practical imaging systems.

**Figure 7:**
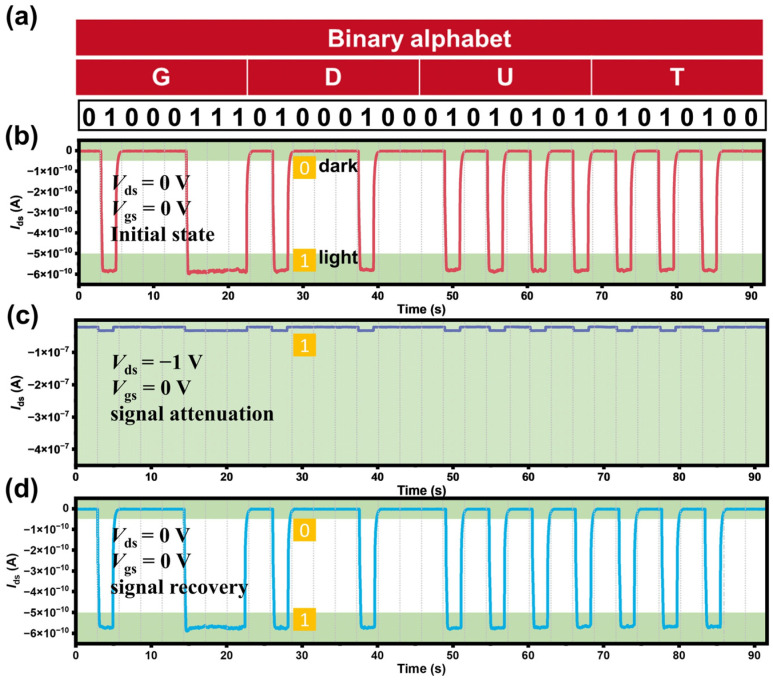
(**a**) Binary alphabets for the letters “G”, “D”, “U”, and “T”. (**b**) Time-dependent source-drain current (*I*_ds_, red line) showing the photovoltaic response of the MoTe_2_/Bi_2_Te_3_ heterojunction at *V*_ds_ = 0 V and *V*_gs_ = 0 V, which corresponds to the binary logic states modulated by laser on/off. The “GDUT” electrical signal of (**c**) attenuation and (**d**) recovery, respectively.

Since the device shows a pronounced photoresponse with a distinct light switching on/off ratio in photovoltaic mode but a smaller light switching ratio when applying external drain bias, it can be considered to be employed in bias-controlled signal modulation as well. Namely, the device demonstrates excellent sensitivity to light on/off at *V*_gs_ = 0 V, enabling secure data transmission in favor of a small drain bias (−1 V). The logic states of “1” and “0” are defined by a current corresponding to the on state (*I*_ds_ > −5 × 10^−10^ A) and the off state (*I*_ds_ < −5 × 10^−11^ A), which are modulated by switching the laser on and off. As shown in Figure 7a, the American Standard Code for Information Interchange (ASCII) code is used to represent “GDUT” in binary format. In optical communication without bias in Figure 7b, the MoTe_2_/Bi_2_Te_3_ heterostructure works in self-powered mode, producing logic states of “1” and “0” modulated by the on and off of 808 nm laser illumination at low optical power (26.4 W/m^2^). To encrypt the data, a bias of *V*_ds_ = −1 V is applied to the device, resulting in both large dark and photocurrent. In this way, the output remains at logic “1” and overwrites the initial “1” and “0” switchable state; therefore, the signal attenuation is realized. Subsequently, withdrawing *V*_ds_ allows signal recovery, with the switchable “1” and “0” states rapidly restored. The fast and reversible signal attenuation and recovery by applying small driven voltage and low-power light realized in the photoresponsive MoTe_2_/Bi_2_Te_3_ heterostructure suggests its great potential in bias-controlled signal modulation.

## 4. Conclusions

In this work, a stacked MoTe_2_/Bi_2_Te_3_ p–n heterojunction is fabricated and systematically studied. It turns out that the device exhibits rectifying behavior in dark and distinct photovoltaic characteristics under illumination with broadband photodetection capabilities from 405 to 1550 nm. Under self-powered mode, the device achieves an impressive responsivity of 1.38 A/W and detectivity of 1.90 × 10^12^ Jones at 532 nm. In the effect of reverse bias of −1 V, the responsivity is promoted to 36.22 A/W with a faster response time of around 32 ms, which is half the time needed at zero bias. Moreover, the device presents good capabilities of optical imaging and bias-controlled signal modulation, evidenced by the high-resolution photocurrent mapping and light-controllable on/off states (signal attenuation by drain bias). Leveraging its favorable photoelectric properties, including good light switching ratio, sensitive self-powered photoresponse, and broadband detection, the MoTe_2_/Bi_2_Te_3_ device is promising for low-power-consumption optical imaging and bias-controlled signal modulation. This work suggests the effective strategy of designing van der Waals heterostructures for future near-infrared detection systems by selecting proper constituent layers that show large light absorption and form suitable band alignment.

## Data Availability

The original contributions presented in this study are included in the article/Supplementary Material. Further inquiries can be directed to the corresponding authors.

## Supplementary Materials

The following supporting information can be downloaded at https://www.mdpi.com/article/10.3390/ma19061270/s1. Figure S1: Transfer curves measured at *V*_ds_ = 2 V of (a) MoTe_2_ and (b) Bi_2_Te_3_, respectively. Figure S2. X-ray photoelectron spectroscopy of (a) MoTe_2_ and (b)Bi_2_Te_3_. The intercept of the X-axis indicates the energy level of the valence-band maximum (VBM) with respect to the Fermi level of the probe having work functions of 4.56 eV. Therefore, the VBM of MoTe_2_ and Bi_2_Te_3_ is −5.07 and −5.05 eV with respect to the vacuum level, respectively. (c) The potential difference between MoTe_2_ and Bi_2_Te_3,_ with an inset of the potential height profile. The calculated band structures of (d) bulk MoTe_2_ and (e) bulk Bi_2_Te_3_, respectively. Figure S3: XPS spectrum of (a) MoTe_2_ and (b) Bi_2_Te_3_, respectively. Table S1. Peak assignment, FWHM, and binding energy of each peak. Figure S4: The Raman spectra of MoTe_2_, Bi_2_Te_3_ and MoTe_2_/Bi_2_Te_3_. Figure S5. AFM image of (a) MoTe_2_ and (b) Bi_2_Te_3_ interface, respectively. Figure S6: The *I*_ds_-*t* response of the MoTe_2_/Bi_2_Te_3_ heterojunction at *V*_ds_ = −1 V in dark and under the irradiation of (a) 405 nm, (b) 635 nm, (c) 808 nm, and (d) 1060 nm lasers, respectively, with different light power intensities. Figure S7: The light-power-dependent responsivity and detectivity under the irradiation of (a) 405 nm, (b) 635 nm, (c) 808 nm, and (d) 1060 nm lasers, respectively. Figure S8: Photocurrent of the heterojunction as a function of incident power density at (a) 405 nm, (b) 635 nm, (c) 808 nm, and (d) 1060 nm laser wavelengths, together with the corresponding linear fitting results. Figure S9: The *I*_ds_-*t* response of the MoTe_2_/Bi_2_Te_3_ heterojunction at *V*_ds_ = 0 V in dark and under the irradiation of (a) 635 nm, (b) 808 nm, and (c) 1310 nm lasers, respectively, with different light power intensities. The light-power-dependent responsivity and detectivity under the irradiation of (d) 635 nm, (e) 808 nm, and (f) 1310 nm lasers, respectively. Figure S10: The *I*_ds_-*t* response of the MoTe_2_/Bi_2_Te_3_ heterojunction at *V*_ds_ = 0 V in dark and under the irradiation of 1550 nm. Figure S11. (a) *I*_ds_-*t* characteristics of MoTe_2_/Bi_2_Te_3_ devices and MoTe_2_ devices, measured at *V*_ds_ = 0 V and *V*_gs_ = 0 V. (b) Optical response time of MoTe_2_ devices. (c) *I*_ds_-*V*_ds_ characteristics of Bi_2_Te_3_ at *V*_gs_ = 0 V. Table S2. Performance of a Self-Powered Photodetector Based on Heterojunctions. Figure references [29,31,33,37,38,39,40,41,42,43,44,45,46,47,48,49,50,51,52,53] are cited in the Supplementary Materials.
