# Supplementary material for: A Broad-Band Self-Powered Photodetector Based on a MoTe2/Bi2Te3 Heterojunction for Optical Imaging and Bias-Controlled Signal Modulation
Source: Materials (Basel). 2026 Mar 23;19(6):1270. doi: 10.3390/ma19061270 (PMC13027777; doi:10.3390/ma19061270)
Supplement: Supplementary file 1 [file materials-19-01270-s001.zip › materials-4158555-supplementary.pdf]

Supplementary Materials

A Broad-band Self-powered Photodetector Based on a $MoTe_2/Bi_2Te_3$ Heterojunction for Optical Imaging and Bias-controlled Signal Modulation

Shaoxiong Du [1], Kunle Li [1], Weijie Li [1], Jiahui Feng [1], Yunwei Sheng [1,*], Lili Tao [1], Zhaoqiang Zheng [1], Wei Song [2], and Yu Zhao [1,*]

[1] Guangdong Provincial Key Laboratory of Functional Soft Condensed Matter, School of Material and Energy, Guangdong University of Technology, Guangzhou 510006, China; dushaoxiong0402@outlook.com (S.D.); qq1060338341@163.com (K.L.); 13169965646@163.com (W.L.); fengjhui@163.com (J.F.); taoll@gdut.edu.cn (L.T.); zhengzhq5@gdut.edu.cn (Z.Z.)

[2] Analysis and Test Center, Guangdong University of Technology, Guangzhou 510006, China; songw@gdut.edu.cn (W.S.)

* Correspondence: ywsheng@gdut.edu.cn (Y.S.); zhaoyu@gdut.edu.cn (Y.Z.)

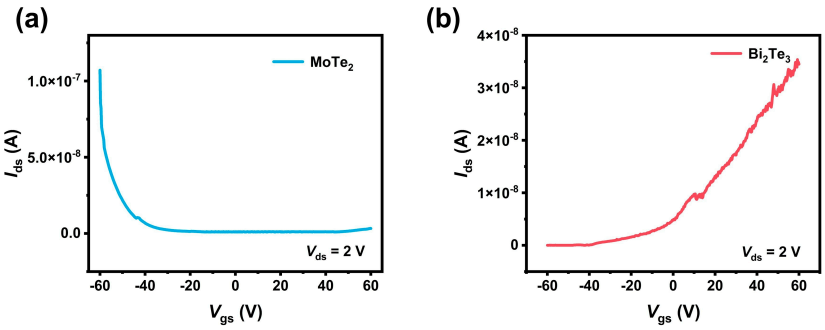

Figure S1. Transfer curves measured at V_{ds} = 2 V of (a) $MoTe_2$ and (b) Bi_2Te_3, respectively.

To demonstrate the conduction type of mechanically exfoliated materials, the wide-range transfer characteristics of the fabricated $MoTe_2$ and Bi_2Te_3 based devices are presented in Figures S1 (a) and (b), respectively. In Figure S1 (a), at a constant drain bias (V_{ds}) of 2 V, the source-drain current (I_{ds}) of the $MoTe_2$ device decreases with increasing positive gate voltage (V_{gs}), confirming its p-type behavior. Similarly, as shown in Figure S1(b), with V_{gs} scanned from -60 V to +60 V at a fixed V_{ds} = 2 V, the I_{ds} of the Bi_2Te_3 device decreases with increasing negative gate voltage, verifying its n-type nature.

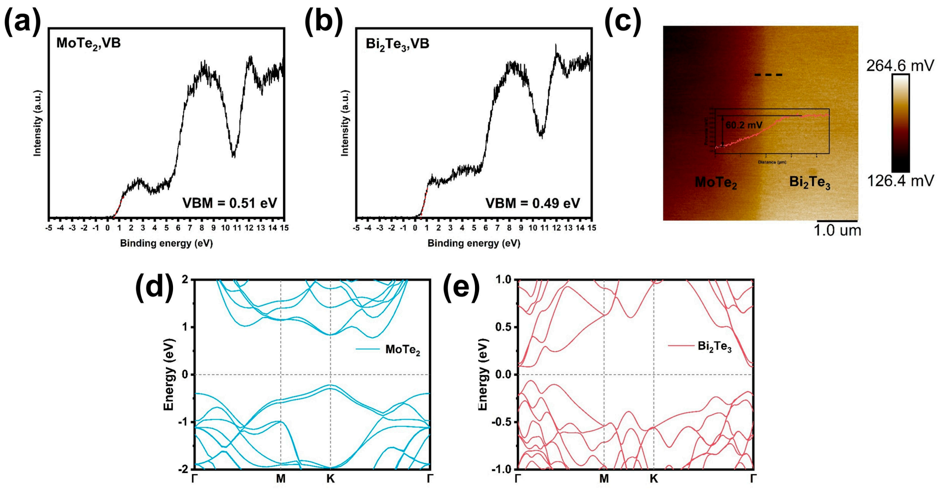

Figure S2. X-ray photoelectron spectroscopy of (a) $MoTe_2$ and (b)Bi_2Te_3. The intercept of X axis indicates the energy level of valence-band maximum (VBM) with respect to Fermi level of the probe having work functions of 4.56 eV. Therefore, the VBM of $MoTe_2$ and Bi_2Te_3 is -5.07 and -5.05 eV with respect to vacuum level, respectively. (c) The potential difference between $MoTe_2$ and Bi_2Te_3 with an inset of the potential height profile. The calculated band structures of (d) bulk $MoTe_2$ and (e) bulk Bi_2Te_3, respectively.

Kelvin probe force microscopy (KPFM) was used to measure the built-in contact potential difference (CPD) at the interface between the $MoTe_2$ flake and the Bi_2Te_3 flake, as shown in Figure S2(c). The surface potential distribution (SPD) of the $MoTe_2$ and Bi_2Te_3 flakes relative to the AFM tip can be expressed by the following equations:

$$eSPD_{MoTe_2} = W_{tip} - W_{MoTe_2} \tag{5}$$

$$eSPD_{Bi_2Te_3} = W_{tip} - W_{Bi_2Te_3} \tag{6}$$

where e is the elementary charge, and W_{MoTe_2}, $W_{Bi_2Te_3}$, and W_{tip} are the work functions of the $MoTe_2$ flake, Bi_2Te_3 flake, and AFM tip, respectively. The Fermi level difference ΔE_f can be calculated using the following equation.

$$\Delta E_f = W_{Bi_2Te_3} - W_{MoTe_2} = eSPD_{MoTe_2} - eSPD_{Bi_2Te_3} \tag{7}$$

As shown in the inset of Figure S2(c), the work function difference between $MoTe_2$ and Bi_2Te_3 is about 60.2 mV. In this work, first-principles calculations were performed based on density functional

theory (DFT), and all calculations were carried out using the Vienna Ab initio Simulation Package (VASP). The electron-ion interaction was described via the projector augmented wave (PAW) method, while the exchange-correlation energy was treated with the Perdew–Burke–Ernzerhof (PBE) functional within the framework of the generalized gradient approximation (GGA). The cutoff energy (ENCUT) for the plane-wave basis set was set to 400 eV, and the energy convergence criterion for electronic self-consistent iterations was set to 10^{-6} eV. Given that the system contains heavy elements whose relativistic effects exert a significant impact on the electronic structure, spin–orbit coupling (SOC) was further introduced to perform accurate electronic structure calculations after the convergence of the self-consistent computation. As shown in the Figure S2 (d) and (e), the calculated band structures of bulk $MoTe_2$ and bulk Bi_2Te_3 are presented. It can be concluded from the figure that both materials possess an indirect bandgap, with bandgap values of 0.99 eV and 0.145 eV, respectively, which are in good agreement with the corresponding bandgap values reported in previous studies [1, 2].

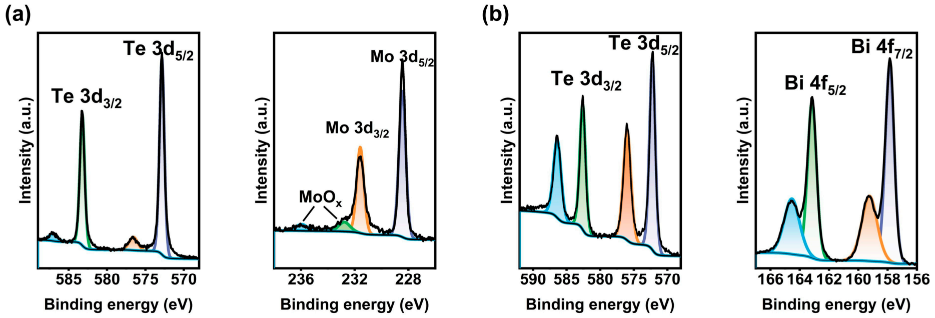

Figure S3. XPS spectrum of (a) $MoTe_2$ and (b) Bi_2Te_3, respectively.

Table S1. Peak assignment, FWHM and binding energy of each peak.

Name	FWHM fit param (eV)	Peak BE (eV)
Te $3d_{3/2}$(Te-O)	1.36	587.01
Te $3d_{3/2}$(Mo-Te)	0.78	583.22
Te $3d_{5/2}$(Te-O)	1.36	576.57
Te $3d_{5/2}$(Mo-Te)	0.78	572.85
Mo $3d_{3/2}$ (Mo-O)	1.02	235.95
Mo $3d_{5/2}$ (Mo-O)	1.02	232.77
Mo $3d_{3/2}$	0.64	231.58
Mo $3d_{5/2}$	0.62	228.45
Te^{4+} (1)	1.41	586.38

Te $3d_{3/2}$	1.06	582.60
Te^{4+} (2)	1.39	575.98
Te $3d/_{5/2}$	1.06	572.22
Bi $4f/_{5/2}$	0.75	163.16
Bi $4f/_{7/2}$	0.75	157.85

Figure S3 and Table S1 present the XPS spectra of $MoTe_2$ and Bi_2Te_3, and the corresponding detailed quantitative data, respectively. Figure S3 (a) shows characteristic peaks of the Mo element near 230 eV in the right panel of the XPS spectrum. Specifically, two dominant peaks at 228.45 eV and 231.58 eV are assigned to Mo^{4+} $3d_{5/2}$ and Mo^{4+} $3d_{3/2}$, respectively. In addition, a distinct characteristic peak at 235.95 eV corresponds to the Mo^{6+} state, forming a characteristic doublet of Mo^{6+} $3d_{5/2}$ and Mo^{6+} $3d_{3/2}$. Notably, the Mo^{6+} $3d_{5/2}$ peak overlaps with the Mo^{4+} $3d_{3/2}$ peak. Through peak fitting, we resolve the Mo^{6+} $3d_{5/2}$ component at 232.77 eV and the Mo^{4+} $3d_{3/2}$ component at 231.58 eV, respectively. This result confirms the existence of Mo-O bonds. The formation of substitutional oxygen defects in the lattice induces hole doping, resulting in hole-dominated p-type carrier transport in the exposed $MoTe_2$ layer, which is in good agreement with the transfer curve of $MoTe_2$ in Figure S1 (a).

In the left panel of Figure S3 (a), the XPS peaks at 572.85 eV and 583.22 eV are attributed to Te $3d_{5/2}$ and Te $3d_{3/2}$ of the Mo-Te bond, respectively, while the peaks at 576.57 eV and 587.01 eV correspond to Te $3d_{5/2}$ and Te $3d_{3/2}$ of the Te-O bond, respectively [3]. In addition, the XPS characteristic peaks at 572.22 eV and 582.6 eV in Figure S3 (b) are assigned to Te $3d_{5/2}$ and Te $3d_{3/2}$, respectively. The extra peaks at 575.98 eV and 586.38 eV originate from the oxidized Te^{4+} species in Bi_2Te_3. The right panel of Figure S3 (b) presents the Bi 4f spectrum of Bi_2Te_3, which exhibits two characteristic peaks at the high-binding-energy end (163.16 eV) and low-binding-energy end (157.85 eV), corresponding to the spin-orbit split Bi $4f_{5/2}$ and Bi $4f_{7/2}$ states, respectively. All the measured peak positions are in excellent agreement with previously reported results in the literature [3, 4].

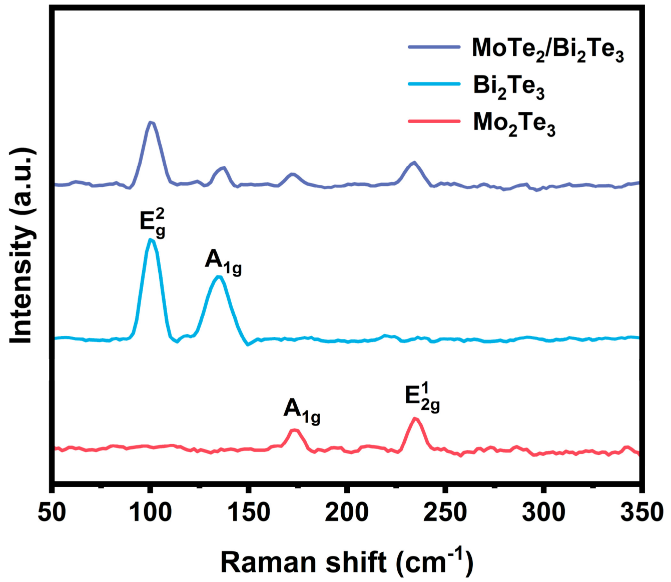

Figure S4. The Raman spectra of $MoTe_2$, Bi_2Te_3 and $MoTe_2/Bi_2Te_3$.

As shown in Fig. S4, The Raman spectrum of pristine Bi_2Te_3 (blue curve) exhibits characteristic Raman peaks at 99.93 cm^{-1} and 134.17 cm^{-1}, corresponding to the E_g^2 and A_{1g} vibrational modes, respectively. Pristine $MoTe_2$ shows distinct vibrational peaks at 173.35 cm^{-1} (A_{1g}) and 234.25 cm^{-1} (E_{2g}^1), respectively.The Raman spectrum of the $MoTe_2/Bi_2Te_3$ heterostructure retains the phonon modes from both constituent materials. Notably, the reduced peak intensity is attributed to Raman quenching across the heterointerface, which is a signature of strong interlayer coupling effects [5]. Raman analysis confirms the phase composition of the $MoTe_2/Bi_2Te_3$ heterostructure, and is in excellent agreement with previously reported spectral data for both materials [6, 7]. It is worth noting that a slight red shift of the A_{1g} peak of $MoTe_2$ is observed. This phenomenon originates from the spontaneous electron transfer from Bi_2Te_3 to $MoTe_2$, resulting in an electron doping effect in $MoTe_2$. This indicates that the constituent materials in the heterostructure are free of contamination after the PVA-assisted dry transfer process [8], demonstrating the formation of a well-defined interface in the van der Waals heterostructure.

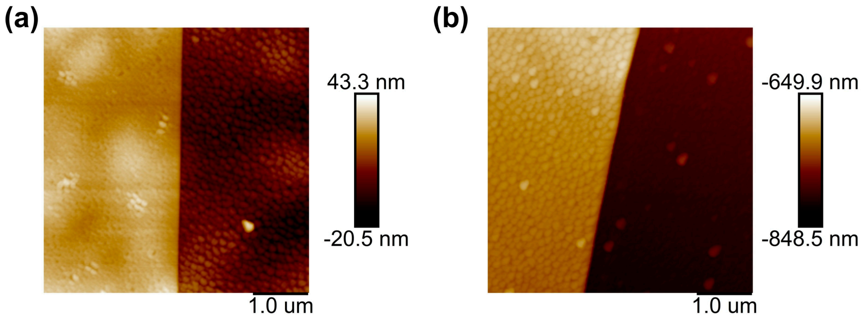

Figure S5. AFM image of (a) $MoTe_2$ and (b) Bi_2Te_3 interface, respectively.

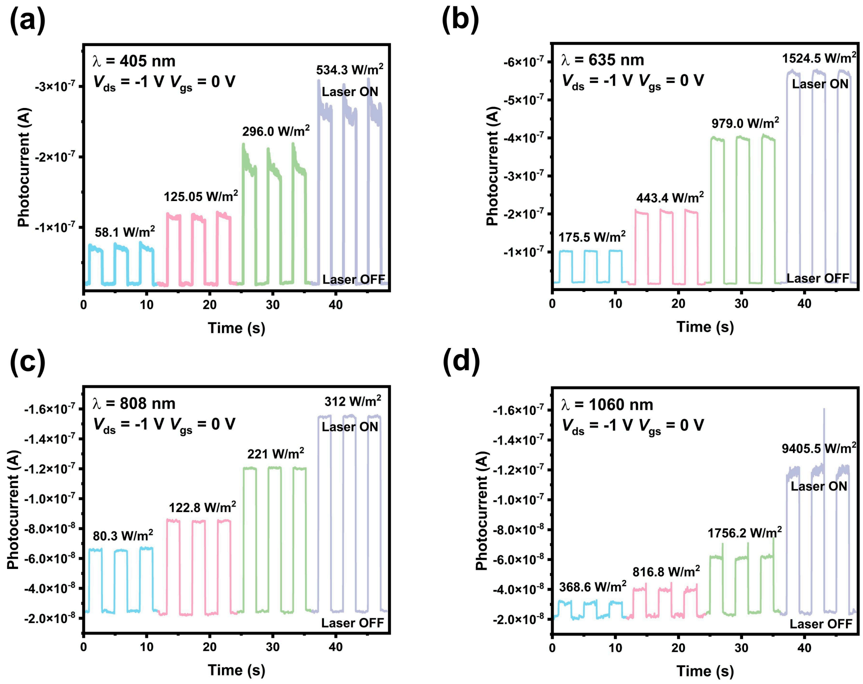

Figure S6. The I_{ds}-t response of the $MoTe_2/Bi_2Te_3$ heterojunction at V_{ds} = -1 V in dark and under the irradiation of (a) 405 nm, (b) 635 nm, (c) 808 nm and (d) 1060 nm laser, respectively, with different light power intensities.

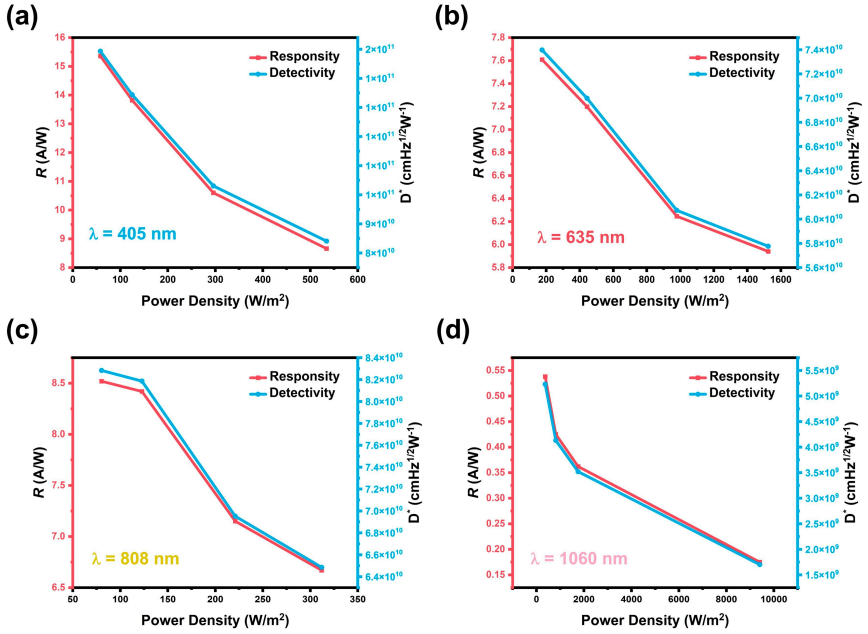

Figure S7. The light-power-dependent responsivity and detectivity under the irradiation of (a) 405 nm, (b) 635 nm, (c) 808 nm and (d)1060 nm laser, respectively.

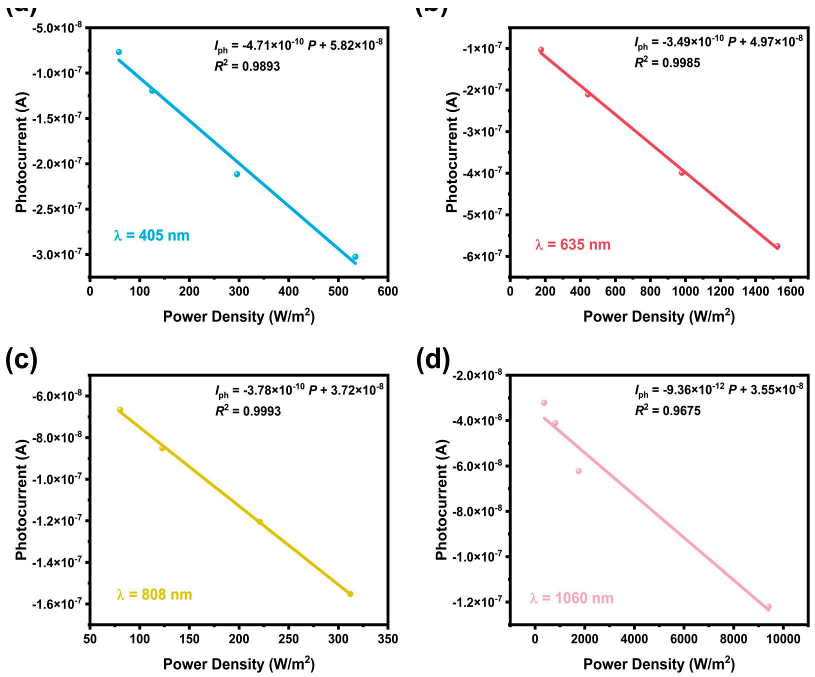

Figure S8.. Photocurrent of the heterojunction as a function of incident power density at (a) 405 nm, (b) 635 nm, (c) 808 nm and (d) 1060 nm laser wavelengths, together with the corresponding linear fitting results.

Take 808 nm laser as an example, by fitting the experimental data, the photocurrent and optical power density satisfy the relation $I_{\text{ph}}(\text{A}) = -3.78 \times 10^{-10}P + 3.72 \times 10^{-8}$ (R^2=0.999, P has units of W/m^2).

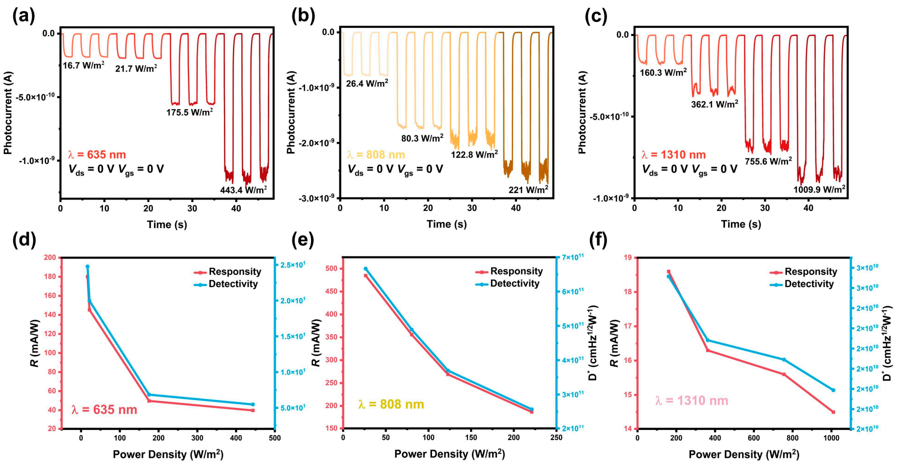

Figure S9. The I_{ds}-t response of the $MoTe_2/Bi_2Te_3$ heterojunction at V_{ds} = 0 V in dark and under the irradiation of (a) 635 nm, (b) 808 nm and (c)1310 nm laser, respectively, with different light power intensities. The light-power-dependent responsivity and detectivity under the irradiation of (d) 635 nm, (e) 808 nm and (f)1310 nm laser, respectively.

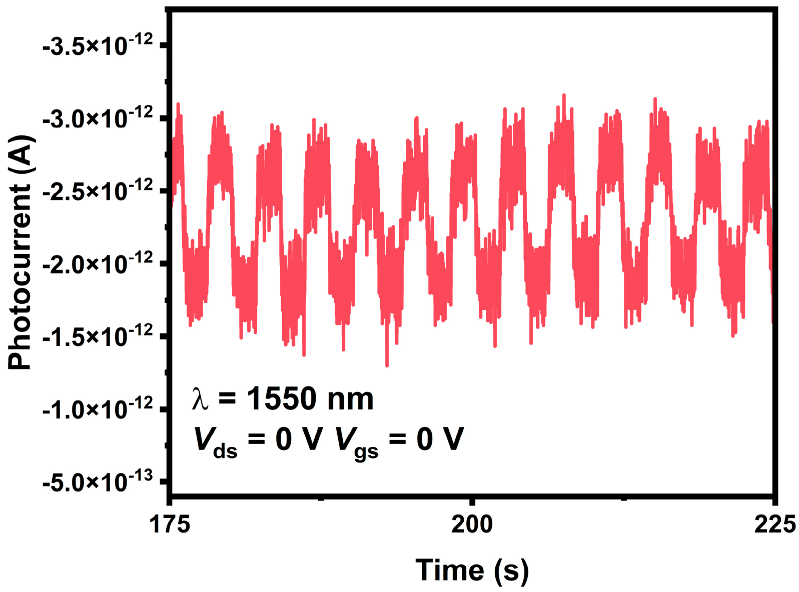

Figure S10. The I_{ds}-t response of the $MoTe_2/Bi_2Te_3$ heterojunction at V_{ds} = 0 V in dark and under the irradiation of 1550 nm.

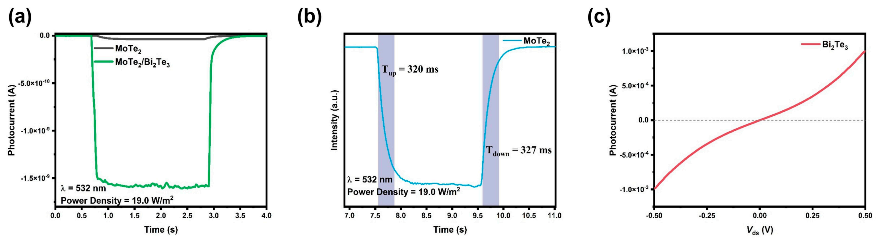

Figure S11. (a) I_{ds} - t characteristics of $MoTe_2/Bi_2Te_3$ devices and $MoTe_2$ devices, measured at $V_{ds} = 0$ V and $V_{gs} = 0$ V. (b) Optical response time of $MoTe_2$ devices. (c) I_{ds}–V_{ds} characteristics of Bi_2Te_3 at $V_{gs} = 0$ V.

To further compare the photoresponse characteristics of the heterojunction device and the pristine single-component $MoTe_2$ device, Figure S11 presents the photocurrent and response speed of the two devices. The results indicate that the photocurrent of the $MoTe_2/Bi_2Te_3$ heterojunction device is 43 times higher than that of the pristine $MoTe_2$ device. As shown in Figure S11 (b), the $MoTe_2$ device exhibits a rise time of 320 ms and a decay time of 327 ms, which are significantly slower than those of the $MoTe_2/Bi_2Te_3$ device (rise time: 77 ms, decay time: 75 ms). Furthermore, as shown in Figure S11 (c), the pristine single-component Bi_2Te_3 device exhibits an ultra-high dark current of approximately 1 mA at $V_{ds} = \pm0.5$ V under zero gate voltage.

Table S2. Performance of a Self-Powered Photodetector Based on Heterojunctions.

Heterojunction	λ (nm)	R_λ (mA/W)	Response / decay time	Spectral response range (nm)	ref
Ga_2O_3/Bi_2Se_3	365	0.175	28 ms/36 ms	254~1000	[9]
$MoTe_2/MoS_2$	915	79	180 μs/202 μs	520~1550	[10]
Bi_2Te_3/Sb_2O_3/p-Si	850	316.5	24.6 ms/25.1 ms	254~1050	[11]
$Gr/WSe_2/MoTe_2$	785	330	6.49 μs/6.22 μs	405~1064	[12]
$Te/Bi_2Te_3/In_2O_3$	850	0.4195	52.8 ms/30.6 ms	850~1050	[2]
$MoTe_2/MoWS_4$	1310	600	21.6 ms/5.8 ms	532~1550	[13]
$SnSe_2/MoTe_2$	532	459	1.16 ms/1.14 ms	405~635	[14]
$GeS_2/MoTe_2$	850	1090	56 ms/64 ms	520~1340	[15]
$MoTe_2$/InSe	405	433.88	99 μs /117 μs	405~980	[16]
ZnSnO/SnSe	365	895	0.9 ms/3.0 ms	365~670	[17]
$SnSe_2/MoTe_2$	520	1470	101 μs /113 μs	520~1550	[18]
2H-$MoTe_2$/1T'-$MoTe_2$	660	80.59	2.4 ms/5.2 ms	405~2200	[19]
MnS/MoS_2	532	1310	20 ms/30 ms	405~635	[20]

$MoTe_2/Bi_2Te_3$	532	1380	32 ms/33 ms	405~1550	This work

1. Wang, F.; Hu, F.; Dai, M.; Zhu, S.; Sun, F.; Duan, R.; Wang, C.; Han, J.; Deng, W.; Chen, W., A two-dimensional mid-infrared optoelectronic retina enabling simultaneous perception and encoding. *nature communications* **2023,** 14, (1), 1938.
2. Zhao, C.; Wang, D.; He, W.; Liu, D.; Cao, J.; Zhang, X.; Liu, S.; Zhang, B.; Pan, J.; Zeng, Z., Photothermal synergistic high-sensitivity self-driven vertical asymmetric Te/Bi2Te3/In2O3 heterojunction near-infrared imaging photodetector. *Chemical Engineering Journal* **2024,** 486, 150183.
3. Qiu, Z.; Liao, J.; Zhang, Y.; Liu, Z.; Wu, Z.; Zheng, H.; Zheng, L.; Zhang, Y., Enhancing Optoelectronic Performance of MoTe2/MoS2 van der Waals Heterostructure Photodiode by Air Annealing. *ACS Applied Electronic Materials* **2025,** 7, (17), 8170-8179.
4. Zhang, C.; Peng, S.; Wei, Y.; Li, C.; Han, J.; Zhou, H.; Jiang, Y.; Wang, J., Photodetectors Based on Vertically Stacked Bi2Te3/WSe2 Heterojunctions for Visible to Near-Infrared Photodetection. *ACS Applied Nano Materials* **2024,** 7, (9), 10565-10572.
5. Che, M.; Wang, B.; Zhao, X.; Li, Y.; Chang, C.; Liu, M.; Du, Y.; Qi, L.; Zhang, N.; Zou, Y., PdSe2/2H–MoTe2 heterojunction self-powered photodetector: Broadband photodetection and linear/circular polarization capability. *ACS nano* **2024,** 18, (44), 30884-30895.
6. Qiu, Z.; Luo, Z.; Chen, M.; Gao, W.; Yang, M.; Xiao, Y.; Huang, L.; Zheng, Z.; Yao, J.; Zhao, Y., Dual-electrically configurable MoTe2/In2S3 phototransistor toward multifunctional applications. *ACS nano* **2024,** 18, (39), 27055-27064.
7. Manikandan, V.; George, K.; Thirumurugan, A.; Govindaraj, T.; Harish, S.; Archana, J.; Navaneethan, M., A Bi2Te3 topological insulator/carbon nanotubes hybrid composites as a new counter electrode material for DSSC and NIR photodetector application. *Journal of Colloid and Interface Science* **2025,** 678, 549-559.
8. Dan, Z.; Yang, B.; Song, Q.; Chen, J.; Li, H.; Gao, W.; Huang, L.; Zhang, M.; Yang, M.; Zheng, Z., Type-II Bi2O2Se/MoTe2 van der Waals heterostructure photodetectors with high gate-modulation photovoltaic performance. *ACS Applied Materials & Interfaces* **2023,** 15, (14), 18101-18113.
9. Han, Y.; Jiao, S.; Jing, J.; Chen, L.; Shi, Z.; Rong, P.; Wang, D.; Gao, S.; He, W.; Wang, J., Vertical heterojunction photodetector with self-powered broadband response and high performance. *Chemical Engineering Journal* **2023,** 477, 147060.
10. Kong, L.; Li, G.; Su, Q.; Tan, X.; Zhang, X.; Liu, Z.; Liao, G.; Sun, B.; Shi, T., Polarization-sensitive, self-powered, and broadband semimetal MoTe2/MoS2 van der Waals heterojunction for photodetection and imaging. *ACS Applied Materials & Interfaces* **2023,** 15, (36), 43135-43144.
11. Wu, W.; He, W.; Ling, D.; Chen, L.; Zhang, Y.; Fan, X.; Bi, Y.; Wang, D.; Wang, J., Enhanced Self‐Powered Photoresponse of a Bi2Te3‐Based Vertical Heterojunction Broadband Photodetector by Carrier Blocking Layer Engineering. *Small* **2025,** 21, (29), 2501484.
12. Wang, S.; Wu, Z.; Ruan, H.; Zheng, L.; Zhang, Y., 2D Gr/WSe2/MoTe2 vertical heterojunction for self-powered photodiode with ultrafast response and high sensitivity. *Journal of Alloys and Compounds* **2024,** 1006, 176379.
13. Pan, Y.; Zhu, L.; Lu, L.; Ou, J. Z.; Zhu, B.; An, C.; Yu, J.; Fang, H.; Dong, M., High Performance and Polarization‐Sensitive Photodetector Enabled by MoTe2/MoWS4 Heterojunction. *Advanced Optical Materials* **2025,** 13, (25), e01281.
14. Li, J.; Wang, D.; Chen, X.; Zhou, Y.; Luo, H.; Zhao, T.; Hu, S.; Zheng, Z.; Gao, W.; Liu, X., Engineering energy bands in 0D–2D hybrid photodetectors: Cu-doped InP quantum dots on a type-III SnSe 2/MoTe 2 heterojunction. *Nanoscale Horizons* **2025,** 10, (5), 922-932.
15. Cai, Y.; Zhu, Y.; Zhang, H.; Jiang, L.; Shen, Y.; Xu, J.; Ding, S.; Zhou, L.; Tao, Z., Gate-Tunable Photovoltaic Behavior and PolarizationSensitive Near-Infrared Photodetector Based on GeS 2/MoTe 2 Heterostructure. *IEEE Photonics Journal* **2025**.
16. He, S.; Feng, P.; Du, Y.; Ma, Y.; Dang, C.; Shan, A.; Zhao, L.; Wei, T. R.; Li, M.; Gao, L., High Performance Self‐Powered and Vis–Infrared Broadband Photodetectors Based on MoTe2/InSe Van der Waals Heterostructure. *Advanced Optical Materials* **2024,** 12, (11), 2302399.

17. Tao, R.; Chen, S.; Wang, F.; Ye, Z.; Pan, X., High performance amorphous ZnSnO/SnSe heterojunction for broad-spectrum self-powered photodetector. *Surfaces and Interfaces* **2025**, 107612.
18. Liu, Z.-G.; Li, X.; Ni, S.; Hu, Z.-Z.; Zhang, Y.-P.; Zhou, Q.; Zhou, H.-L.; Liu, C.-l.; Zhang, X.-Y.; Zhang, T., Type-III SnSe2/MoTe2 van der Waals heterojunction for room-temperature self-powered broadband photodetection. *Infrared Physics & Technology* **2025**, 106135.
19. Wang, D.; Li, H.; Liu, J.; Qin, Y.; Zhao, J.; Hou, P., Asymmetric schottky contacts enhanced two-dimensional heterojunctions for self-powered broadband and polarization-sensitive photodetection. *ACS Applied Materials & Interfaces* **2025,** 17, (22), 33089-33097.
20. Li, K.; Xie, C.; Chen, T.; Li, W.; Du, S.; Yang, Y.; Tao, L.; Zheng, Z.; Feng, X.; Zhao, Y., High-Performance Self-Powered Photodetectors Based on the MnS/MoS2 Heterojunction with Smooth Interfacial Band Bending. *The Journal of Physical Chemistry C* **2024,** 128, (51), 21730-21738.
